# Integrated transcriptomic and metabolite analysis reveals the role of AktADT8 in the biosynthesis of phenylalanine and its derivatives in *Akebia trifoliata*

**DOI:** 10.1002/tpg2.70342

**Published:** 2026-11-13

**Authors:** Jie Li, Ju Guan, Hao Liu, Rui Han, Huai Yang, Xiaoxiao Yi, Chen Chen, Feiquan Tan, Wanyun Peng, Zhengwei Xie, Peigao Luo

**Affiliations:** ^1^ Rice Research Institute Sichuan Agricultural University Chengdu China; ^2^ Cuisine Science Key Laboratory of Sichuan Province Sichuan Tourism University Chengdu China; ^3^ Provincial Key Laboratory for Plant Genetics and Breeding, College of Agronomy Sichuan Agricultural University Chengdu China; ^4^ Dazhou Academy of Agricultural Sciences, Vocational Education Zone Dazhou China

## Abstract

Arogenate dehydratase (ADT) is a key enzyme in plant phenylalanine (Phe) biosynthesis, providing precursors for protein and phenolic compounds. *Akebia trifoliata* is valued as a medicinal, fruit, and ornamental plant because of its abundance of Phe and derivatives. Among the 373 metabolites identified previously, 135 were phenolic compounds. Furthermore, analysis of 153 transcriptomic datasets from 53 samples across nine BioProjects revealed that *AktADT3* and *AktADT8* exhibited obvious stage‐specific expression patterns. A total of 758 mutation sites were detected in *AktADTs*, with *AktADT8* displaying a markedly higher proportion of synonymous variants. Co‐expression analysis indicated that *AktADT* transcript abundance correlated significantly with the levels of 124 metabolites and 150 enzyme‐encoding genes across five flavonoid biosynthesis pathways, with *AktADT8* uniquely showing broad positive correlations across all pathways. Overexpression of *AktADT6* and *AktADT8* in *Arabidopsis* transformant lines presented significantly increased contents of Phe and flavonoids. Moreover, *AktADT8* was uniquely characterized by two transcription start sites, and binding prediction suggested that *MYB105* (where MYB is v‐myb avian myeloblastosis viral oncogene homolog) could bind to the promoter region of *AktADT8*, whereas *MYB21* could bind to the promoter regions of both *AktADT8* and *AktADTs8*, thereby regulating their transcriptional activity. These findings highlight *AktADT8* as a key candidate gene involved in Phe metabolism in *A. trifoliata*.

## Plain Language Summary

Phenylalanine (Phe) is an important plant building block used for proteins and compound biosynthesis, including flavonoids and other natural chemicals with medicinal value. *Akebia trifoliata* is widely used as a fruit, ornamental, and medicinal plant because it accumulates high levels of these compounds. This study investigated a group of genes called *Arogenate dehydratase (ADT)*, which control the production of Phe. Analysis of previously published datasets showed that *AktADT8* is a key gene involved in Phe synthesis through both pathways in *A. trifoliata*. It is active at specific developmental stages and is closely associated with flavonoid production. Overexpression of *AktADT8* in *Arabidopsis* increased Phe and flavonoid levels. Moreover, *MYB105* (where MYB is v‐myb avian myeloblastosis viral oncogene homolog) could bind to the *AktADT8* promoter, whereas *MYB21* could bind to the promoters of both *AktADT8* and *AktADTs8*. These results identify *AktADT8* as a key gene involved in the biosynthesis of important natural compounds in *A. trifoliata*.

**Abbreviations**
AbA: aureobasidin A
ADT: arogenate dehydratase
CDS: coding DNA sequence
DEL: deletion
GUS: β‐glucuronidase
INS: insertion
LUC: luciferase
Kan: kanamycin
MYB: v‐myb avian myeloblastosis viral oncogene homolog
PAC: PDT activity‐conferring
PC: principal component
PCR: polymerase chain reaction
PDT: prephenate decarboxylase/dehydratase
Phe: phenylalanine
Rif: rifampicin
REN: Renilla luciferase
SNP: single‐nucleotide polymorphism
TFBS: transcription factor‐binding site
TF: transcription factor
TSS: transcription start site
UTR: untranslated region
Y1H: yeast one‐hybrid

## 1 INTRODUCTION

During their evolution, plants have developed specific secondary metabolites as key adaptive molecules to resist environmental and biotic stresses (Dixon & Dickinson, 2024). Based on their chemical structures, plant secondary metabolites are generally classified into three categories: terpenoids, phenolics, and alkaloids, among which phenolics represent the most abundant class of plant secondary metabolites (Zhao et al., 2023). To date, >8000 natural phenolics compounds and their derivatives have been identified in plants (Zhu et al., 2025). These metabolites play important roles in plant growth and development (Wu et al., 2025), defense responses (Ma et al., 2024), and ornamental traits (L. Wang et al., 2023). They are also widely exploited in various industrial sectors, including pharmaceuticals (W. Xu, Lin, et al., 2025a), food processing (X. Chen et al., 2024), cosmetics (Serra et al., 2023), and materials science (He et al., 2025). Phenylalanine (Phe) serves as a central precursor for phenylpropanoid‐derived polyphenols, such as lignin, flavonoids, and anthocyanins, making its biosynthesis a key regulatory node in plant secondary metabolism (Dong & Lin, 2021; Skaliter et al., 2022).

In plants, only two pathways for the synthesis of Phe have been reported, namely, the arogenate pathway and the phenylpyruvate pathway (Qian et al., 2019). The arogenate pathway in chloroplasts is usually viewed as the main synthesis pathway because it is widely conserved in plants (Maeda et al., 2010). In this pathway, prephenate is first transformed into arogenate by prephenate aminotransferase, a molecule, which is then decarboxylated/dehydrated to Phe by arogenate dehydratase (ADT). In contrast, the phenylpyruvate pathway is localized in the cytosol, where prephenate is first transformed into phenylpyruvate by prephenate decarboxylase/dehydratase (PDT) and then phenylpyruvate is further transformed to produce Phe by phenylpyruvate aminotransferase (Tzin et al., 2009). Although this pathway has been identified in microorganisms (Berry et al., 1987; Fischer et al., 1993), it has been identified in only a small number of plant species, such as *Pinus pinaster* (El‐Azaz et al., 2016), *Oryza sativa* (Yamada et al., 2008), *Arabidopsis thaliana* (Q. Chen et al., 2016), *Petunia hybrid* (Qian et al., 2019), and *Akebia trifoliata* (Guan et al., 2024). Thus, to date, the phenylpyruvate pathway has generally been considered a compensatory pathway in plants.

ADT and PDT are key enzymes in the arogenate and phenylpyruvate synthesis pathways, respectively (Tzin & Galili, 2010). Notably, although genes encoding ADT enzymes have been identified in plant genomes, no genes encoding PDT enzymes have been reported in any plant species. However, some ADT enzymes, such as *AtADT1*, *AtADT2*, and *AtADT6* in *A. thaliana* (Cho et al., 2007); *PpADT‐B*, *PpADT‐C*, *PpADT‐D*, and *PpADT‐G* in *P. pinaster* (El‐Azaz et al., 2016); *PhADT2* and *PhADT3* in *P. hybrida* (Qian et al., 2019); and *AktADT1*, *AktADT4*, *AktADT7*, *AktADT8*, and *AktADTs8* in *A. trifoliata* (Guan et al., 2024), have PDT activity in addition to ADT activity and can catalyze the reaction from prephenate to phenylpyruvate. These dual‐function ADTs typically contain a conserved PDT activity‐conferring (PAC) domain composed of 22 amino acid residues, some of which are highly conserved (El‐Azaz et al., 2016). In addition, some *ADT* genes likely contain multiple different transcription start sites (TSSs) (Qian et al., 2019). In fact, *ADTs* can be divided into three types, α lacking a PAC domain, β containing an Ala^314^ and Val^317^ combination domain, and γ containing a Val^314^ and Ala^317^ combination domain. Furthermore, extensive analysis of 70 species, including of three basal angiosperms, 22 monocots, 10 basal eudicots, and 35 core eudicots, revealed that α *ADTs* are only found in some plant species, while both β and γ ADTs were identified in all plant species analyzed (Li et al., 2024). Various studies have shown that β ADTs are active only in the presence of low Phe levels, whereas γ ADTs still exhibit strong activity in the presence of high Phe levels (Q. Chen et al., 2016; El‐Azaz et al., 2022; Guan et al., 2024; Li et al., 2024; Qian et al., 2019). This finding indicates that the phenylpyruvate pathway for Phe synthesis could form an “essential” pathway rather than just serving as an “alternative” pathway and could be a parallel evolved pathway of the arogenate pathway.

*A. trifoliata* is a perennial woody climbing vine widely distributed across East Asia, Europe, and North America. Commercially, *A. trifoliata* has been used as a medicinal material in China for over 2000 years because of its richness in bioactive secondary metabolites, especially Phe‐derived compounds such as flavonoids (Higuchi & Kawasaki, 1976; P. Huang et al., 2022; S. F. Zhong, Guan, et al., 2022). More recently, *A. trifoliata* has gained popularity as an emerging fruit with a unique flavor because of its high content of aromatic amino acids such as Phe derived from the biosynthetic pathway (Guan et al., 2022; S. F. Zhong, Guan, et al., 2022). In terms of theoretical research, *A. trifoliata* is also an ideal species because of its basal position within the eudicot clade as the evolutionary bridge between basal angiosperms and core eudicots (Zahn et al., 2010), its short juvenile stage (S. Yang et al., 2023), unique flower structure (S. F. Zhong et al., 2024), ability to cross‐pollinate (Matsumoto et al., 2022), and small genome size (S. F. Zhong, Li, et al., 2022). A recent study showed that the ADT family consists of eight members in *A. trifoliata*. Among them, AktADT4, AktADT7, and AktADT8 have moonlighting PDT activity. Notably, only AktADT8 belongs to the γ type and contains two TSSs, AktADT8 and AktADTs8, which are predicted to encode proteins localized in the chloroplast and cytosol, respectively (Guan et al., 2024; Li et al., 2024). Despite these intriguing features, how *AktADT8* contributes to Phe biosynthesis, metabolic flux partitioning, and downstream polyphenol accumulation in *A. trifoliata* remains largely unknown.

In this study, all published metabolite and transcriptomic datasets of *A. trifoliata* were collected. Published metabolites were classified to determine the abundance and composition of phenolic compounds. Available transcriptomic datasets were subsequently analyzed to characterize the expression patterns and genetic variation of *AktADT* genes. The functions of *AktADT* genes were further investigated by overexpression in *Arabidopsis*. Finally, candidate *AktADTs* and regulatory factors involved in polyphenol biosynthesis were identified. These results provide new insights into the functional diversification of *ADTs* and highlight *AktADT8* as a key candidate gene involved in Phe‐derived secondary metabolism.

## 2 MATERIALS AND METHODS

### 2.1 Statistical analysis of publications related to *A. trifoliata*

Published studies on *A. trifoliata* were identified by searching for the keyword “*Akebia trifoliata*” in the Web of Science Core Collection database (https://webofscience.clarivate.cn/wos, accessed on March 20, 2025) (Table S1). Metabolite information was extracted from the results sections of these publications; all reported metabolites associated with *A. trifoliata* were statistically compiled, and duplicate data were removed and citations merged, as detailed in Table S2. The collected metabolites were classified into primary metabolites, secondary metabolites, and other compounds. Secondary metabolites were further categorized into polyphenols, terpenoids, alkaloids, and other secondary metabolites. ArcGIS (v10.8) was used to visualize the geographical distribution of the plant samples to obtain the available transcriptomic data.

### 2.2 The source of the transcriptome data

In this study, a total of 153 transcriptomic datasets representing 51 samples (three biological replicates per sample) were collected from nine public BioProjects of *A. trifoliata* (Table 1; Table S3). The five samples in PRJNA685604 were collected from seeds in Huaihua City, Hunan Province, in May, June, July, August, and September (May, June, July, August, and September), respectively (H. Huang et al., 2021). The five samples in PRJNA792843 were collected from seeds in Yuanjiang City, Hunan Province, at 120, 135, 165, 180, and 195 days after anthesis (F, S, T, U, and I), respectively (Y. C. Zhong, Zhao, et al., 2022). The three samples in PRJNA884501 were collected from seeds in Guiyang City, Guizhou Province, at 30, 50, and 70 days after anthesis (30 DAF, 50 DAF, and 70 DAF), respectively (H. J. Liu et al., 2023). The 12 samples in PRJNA671772 were collected from the peel (TP1, TP2, TP3, and TP4), flesh (TF1, TF2, TF3, and TF4), and seeds (TS1, TS2, TS3, and TS4) of plants at four different stages (young, enlargement, coloring, and maturity) in Chengdu City, Sichuan Province (S. F. Zhong, Guan, et al., 2022). The three samples in PRJCA014987 were collected from Chengdu City, Sichuan Province, and represented the peel tissues of 24 healthy fruits (RP), 50 susceptible fruits with many spot types and disease severity scores of 4–5 (SP), and the sequenced cultivar Shusen 1 used as the genome reference control (CP) (H. Yang, Wang, et al., 2024). The three samples in PRJNA524995 were collected from the peels at 10‐day intervals during the ripening period (September 18, September 28, and October 8, 2018) in Changsha City, Hunan Province, including the non‐cracking stage (PS), initial cracking stage (PM), and total cracking stage (PL) (Niu et al., 2020). The 12 samples in PRJCA045110 were collected from seeds at the yellow stage (Y), veraison stage (V), and maturity stage (M) from four materials (L2127, H543, L2167, and H701); L2127 and L2167 were obtained from Zhaotong City, Yunnan Province; H543 was obtained from Liping City, Guizhou Province; and H701 was obtained from Ningdu City, Jiangxi Province (Han et al., 2025). The three samples in PRJNA1098036 were obtained from Honghe City, Yunnan Province, and leaves were collected under different treatments, including saline‐alkali stress (SAS), saline‐alkali stress and metformin (SAS + M), and distilled water (Control) treatments (K. Wang, Tao, et al., 2024). The five samples in PRJNA857910 were collected from whole fruits in Lu'an City, Anhui Province, from early May to early September (YZZ05, YZZ06, YZZ07, YZZ08, and YZZ09) (Fang et al., 2024).

**TABLE 1:** Sources of the samples and basic information on the *Akebia trifoliata* transcriptome.

Number	BioProject number	Source	Sample ID	Tissues	Number	BioProject number	Source	Sample ID	Tissues
1	PRJNA1098036 (K. Wang, Tao, et al., 2024)	Yunnan Honghe	SAS + M	Leaf	27	PRJNA671772 (S. F. Zhong, Guan, et al., 2022)	Sichuang Chengdu	TP1	Peel
2			SAS	Leaf	28			TP2	Peel
3			Control	Leaf	29			TP3	Peel
4	PRJCA045110 (Han et al., 2025)	Yunnan Zhaotong	L2127‐Y	Seed	30			TP4	Peel
5			L2127‐V	Seed	31			TS1	Seed
6			L2127‐M	Seed	32			TS2	Seed
7		Guizhou Liping	H543‐Y	Seed	33			TS3	Seed
8			H543‐V	Seed	34			TS4	Seed
9			H543‐M	Seed	35			TF1	Flesh
10		Yunnan Zhaotong	L2167‐Y	Seed	36			TF2	Flesh
11			L2167‐V	Seed	37			TF3	Flesh
12			L2167‐M	Seed	38			TF4	Flesh
13		Jiangxi Ningdu	H701‐Y	Seed	39	PRJNA792843 (Y. C. Zhong, Zhao, et al., 2022)	Hunan Wanjiang	F	Seed
14			H701‐V	Seed	40			S	Seed
15			H701‐M	Seed	41			T	Seed
16	PRJCA014987 (H. Yang, Wang, et al., 2024)	Sichuang Chengdu	CP	Peel	42			U	Seed
17			RP	Peel	43			I	Seed
18			SP	Peel	44	PRJNA685604 (H. Huang et al., 2021)	Hunan Huaihua	May	Seed
19	PRJNA884501 (H. J. Liu et al., 2023)	Guizhou Guiyang	30 DAF	Seed	45			June	Seed
20			50 DAF	Seed	46			July	Seed
21			70 DAF	Seed	47			Aug	Seed
22	PRJNA857910 (Fang et al., 2024)	Anhui Lvan	Y5	Fruit	48			Sep	Seed
23			Y6	Fruit	49	PRJNA524995 (Niu et al., 2020)	Hunan Changsha	PS	Peel
24			Y7	Fruit	50			PM	Peel
25			Y8	Fruit	51			PL	Peel
26			Y9	Fruit					

Abbreviations: DAF, days after anthesis; M, metformin; SAS, saline‐alkali stress.

### 2.3 Sequencing analysis of raw transcriptome data

Among the nine BioProjects, seven (PRJNA) were retrieved from the National Center for Biotechnology Information (https://www.ncbi.nlm.nih.gov/), whereas the remaining two (PRJCA) were obtained from the National Genomics Data Center (CNCB‐NGDC) (https://www.cncb.ac.cn/). The raw transcriptome sequencing data (.sra files) were downloaded using the prefetch command and converted to fastq files using the fastq‐dump command of Sra Toolkit (v1.19.2) (https://github.com/ncbi/sra‐tools). Trimmomatic (v0.39) (Bolger et al., 2014) was used to obtain the clean reads by removing adaptor sequences and low‐quality bases with the following parameters: ILLUMINACLIP:TruSeq3‐PE.fa:2:30:10:8:true, SLIDINGWINDOW:5:20, LEADING:3, TRAILING:3, and MINLEN:50. FastQC (v0.12.1) (https://github.com/s‐andrews/FastQC) was used to assess the quality of raw and clean sequencing reads, including per‐base sequence quality distribution, GC content, and adapter contamination.

HISAT2 (v2.2.1) (Zhang et al., 2021) was used to align the clean reads with the assembled reference genome of *A. trifoliata* (S. F. Zhong, Li, et al., 2022), generating Sam files (.sam) and calculating the mapping rates. SAMtools (v1.18) (Danecek et al., 2021) was used to convert Sam files into Bam files and sort them according to chromosome coordinates. StringTie (v2.2.1) (Shumate et al., 2022) was used to estimate gene expression levels as fragments per kilobase of transcript per million mapped reads (FPKM) using default parameters and to merge transcripts from multiple samples using the –merge option. gffcompare (v0.12.6) (Pertea G & Pertea M, 2020) was used to predict to compare assembled transcripts and identify novel transcript models for each sample. Finally, TBtools (v2.310) (C. Chen et al., 2023) was used to visually analyze the correlations among the transcriptomic expression profiles, principal component analysis results and heatmaps.

### 2.4 Analysis of mutations in the transcriptome

The mpileup subcommand of BCFtools (v1.19) (Danecek et al., 2021) was used to identify transcriptome‐derived variant sites from aligned Bam files against the reference genome of *A. trifoliata* (S. F. Zhong, Li, et al., 2022). The call and merge subcommands of BCFtools were subsequently used to identify variant sites and merge variant data from all samples, respectively. The filter subcommand of BCFtools was used to filter low‐quality variant sites based on the following criteria: genotype quality <2, haplotype quality <10, and mutation sites with quality <10. The identified variant sites were annotated using SnpEff (v5.2a) (Cingolani et al., 2012). A genetic distance matrix was constructed using PLINK (v1.90) (Purcell et al., 2007). Phylogenetic trees were constructed and visualized in R (v4.4.1) using the aid of the ape and phangorn (Schliep, 2011) packages. The neighbor‐joining method with 1000 bootstrap replicates was applied to increase the robustness of the tree topology (Galesloot et al., 2014).

### 2.5 Association analysis and analysis of cis‐acting elements in *AktADT8*

The correlations between the expression of *AktADT8* and the reported flavonoid compounds, flavonoid biosynthetic enzyme‐encoding genes, and transcription factors (TFs) (H. Yang, Zhong, et al., 2024) were analyzed using SPSS (v27). The TBtools plugin Fasta Extract or Filter was used to extract the 2000 bp upstream promoter region of *AktADT8*. The PlantCARE database (http://bioinformatics.psb.ugent.be/webtools/plantcare/html/) was used to predict cis‐acting elements in the *AktADT8* promoter. The transcription factor‐binding sites (TFBSs) in *AktADT8* promoter were predicted using the Plant TF binding motif and FIMO binding motif scan plugins in TBtools. The co‐expression network was visualized using Cytoscape (v3.8.0) (Shannon et al., 2003). AlphaFold (https://alphafoldserver.com/) (Abramson et al., 2024) was used to predict the interaction between protein and promoter, and PyMOL (v3.0.3) was used to visualize the predicted results.

### 2.6 Analysis of the effects of *AktADT* overexpression in *A. thaliana*

The coding DNA sequences (CDSs) of *AktADT1‐8* were obtained from the published *A. trifoliata* reference genome of cultivar Shusen1 (S. F. Zhong, Li, et al., 2022) and cloned from leaves of the same cultivar, which was cultivated at the Germplasm Nursery of Chongzhou Research Station, Sichuan Agricultural University, Sichuan, China (103.66°E, 30.56°N). The cloned *AktADT1‐8* sequences were inserted into the KpnI‐ and XbaI‐linearized pCAMBIA1305‐GUS vector (Bomed Biotechnology) through homologous recombination. The cloning primers and homologous recombination primers are listed in Table S4. The recombinant plasmids were subsequently introduced into *Agrobacterium tumefaciens* GV3101 (Daling Biotechnology) by the heat‐shock method, and the transformants were selected on YEP plates (50 µg/mL kanamycin [Kan] + 25 µg/mL rifampicin [Rif]) and incubated at 28°C for 2–3 days. A single colony was picked and inoculated in 2 mL of YEP‐resistant liquid medium (Kan + Rif) for culture. Positive clones were confirmed by polymerase chain reaction (PCR) and subsequently transferred into 50 mL of YEP liquid medium (Kan + Rif) and cultured with shaking at 28°C until the liquid OD_600_ reached 0.8‐1.0. The bacterial pellet was collected by centrifugation at 5000 rpm for 10 min at 4°C, resuspended in a 5% sucrose solution containing 2% surfactant silwetL‐77, and adjusted to an OD_600_ of 0.8‐1.0. The inflorescences of *A. thaliana* (Col‐0) were immersed in the bacterial suspension for 1.5‐2.0 min and cultured in the dark for 10 h. The plants were subsequently returned to a growth chamber under a 16‐h light/8‐h dark photoperiod at 26°C until seed maturation. T1 seeds were collected after 3–4 weeks and germinated on 1/2 Murashige and Skoog medium supplemented with 40 µg/mL hygromycin for selection. When the roots reached approximately 1–2 cm, *A. thaliana* seedlings were placed in 1.5 mL microcentrifuge tubes containing β‐glucuronidase (GUS) staining solution (Solarbio, Beijing, China), incubated at 37°C in the dark for 6 h, and then destained with ethanol, and the GUS staining results were photographed. After the plants matured, plant DNA was extracted, and the presence of the transgene was confirmed by PCR amplification using AktADT1‐8F/AktADT1‐8R primers. Positive transformants were transplanted into soil and grown under a 16‐h light/8‐h dark photoperiod at 26°C to obtain T2. The T3 generation culture follows the same procedure. Eighteen independent homozygous T3 lines were generated for each construct, and lines were used for subsequent phenotypic and biochemical analyses. Non‐transgenic wild‐type *A. thaliana* (Col‐0) served as the control.

### 2.7 Determination of Phe and total flavonoid contents

At the rosette stage (24 days after germination), the leaves were collected from wild‐type and *Arabidopsis* overexpressed lines, and three biological replicates and two technical replicates were analyzed for each treatment. The Phe content was determined according to the method described by S. F. Zhong, Guan, et al. (2022). Briefly, 2 g of leaf tissue was extracted with 2 mL of 0.02 mol/L hydrochloric acid by ultrasonication for 30 min, followed by incubation at 4°C for 2–4 h. After centrifugation, 750 µL of the supernatant was transferred to a new tube and mixed with 250 µL of 10% sulfosalicylic acid. The mixture was incubated at 4°C for 1 h and then filtered through a 0.45‐µm membrane filter before analysis. Phe was analyzed using an amino acid analyzer (L‐8900; Hitachi Co., Japan) equipped with a chromatographic column (855‐4507; Hitachi Co., Japan). Separation was performed using a lithium buffer gradient elution program, and detection was carried out at 570 and 440 nm. The total flavonoid content was determined according to the method described by Guan et al. (2022). 2 g of leaf tissue was extracted with 10 mL of 70% ethanol by ultrasonication for 30 min, and the extracted volume was adjusted to 50 mL with 70% ethanol. A 2 mL aliquot of the extract was sequentially mixed with 2 mL of 5% NaNO_2_, 2 mL of 10% AlCl_3_, and 6 mL of 1 M NaOH at 6‐min intervals. After incubation for 10 min, the absorbance was measured at 510 nm using a spectrophotometer (UV‐2401, Shimadzu Co., Japan). A calibration curve was generated using rutin as the standard, and the total flavonoid content was calculated accordingly.

### 2.8 Dual‐luciferase assay and yeast one‐hybrid assay

The CDSs of *MYB21* and *MYB105* (where MYB is v‐myb avian myeloblastosis viral oncogene homolog), as well as the promoter fragments containing the predicted core promoter elements surrounding TSS1 of *AktADT8* (−150 to +150 bp) and TSS2 of *AktADTs8* (−50 to +230 bp), were cloned from the leaves of *A. trifoliata* ‘Shusen1.’ The CDSs of *MYB21* and *MYB105* were inserted into the pCAMBIA1301‐35S vector (Bomed Biotechnology) to generate effector constructs, whereas the promoter fragments of *AktADT8* and *AktADTs8* were cloned into the pGreenII0800‐LUC vector (NovoPro Bioscience, Shanghai, China) to generate reporter constructs. The recombinant constructs were introduced into *A. tumefaciens* GV3101 competent cells and cultured until the OD_600_ reached 0.8‐1.0. The bacterial cells were collected, washed, and resuspended using infiltration buffer containing 10 mM MgCl_2_, 10 mM 2‐Morpholinoethanesulfonic Acid, and 200 µM acetosyringone to an OD_600_ ranging from 0.6 to 1.0. Three days after infiltration, the leaves were sprayed with 1 mM D‐luciferin sodium solution, and the luminescence signals were recorded using a plant in vivo imaging system (GelView6000PLUS). The activities of firefly luciferase (LUC) and Renilla luciferase (REN) were quantitatively measured using a Dual‐Luciferase Reporter Assay Kit (Yeasen Biotechnology, Shanghai, China) according to the manufacturer's instructions. The LUC/REN ratio was used as an indicator of promoter activity. Relative activation levels were calculated by comparing the LUC/REN ratios of samples co‐transformed with effector and reporter constructs with those of the empty vector control. Statistical significance was evaluated using Student's *t*‐test.

The full‐length CDSs of *MYB21* and *MYB105* were cloned into the pGADT7 vector (Coolaber, Beijing, China) to generate the effector constructs pGADT7‐MYB21 and pGADT7‐MYB105. The promoter fragments of AktADT8 and AktADTs8 (AktADT8pro and AktADTs8pro) were inserted into the pAbAi vector (Coolaber) to generate the bait constructs pAbAi‐AktADT8pro and pAbAi‐AktADTs8pro. Yeast strain Y1HGold competent cells (Daling Biotechnology) carrying the recombinant pAbAi plasmids were cultured on SD/−Leu medium (Coolaber) containing aureobasidin A (AbA, Coolaber) to evaluate promoter auto‐activation. The cell suspension concentrations were adjusted to four gradients (10^°^, 10^−^^1^, 10^−^^2^, and 10^−^^3^) to select the optimal concentration. The suspensions were spotted onto SD/−Ura medium (Coolaber) containing different AbA concentrations (0, 50, 100, 200, 300, 600, and 900 ng/mL) to determine the optimal AbA concentration for promoter activity assays. Finally, the four combinations (pGADT7‐*MYB21* + pAbAi‐*AktADT8pro*, pGADT7‐*MYB105* + pAbAi‐*AktADT8pro*, pGADT7‐*MYB21* + pAbAi‐*AktADTs8pro*, and pGADT7‐*MYB105* + pAbAi‐*AktADTs8pro*) were spotted onto SD/−Ura/−Leu medium (Coolaber) containing the optimal AbA concentration. All primers are listed in Table S1.

## 3 RESULTS

### 3.1 Meta‐analysis of metabolites present in *A. trifoliata* reported in the literature

A total of 132 publications related to *A. trifoliata* were retrieved, including 128 research articles and four reviews (Table S1). Among the research articles, 114 studies used materials collected from China, nine from Japan, one from South Korea, and one from the Korean Peninsula. The numbers of references to “*A. trifoliata*” in the abstracts, titles, and keywords were 122, 106, and 99, respectively (Table S1). A total of 2347 metabolites were counted from 42 (31.82%) of 132 publications surveyed. Among these metabolites, 1974 metabolites were reported in only one publication, whereas 373 were reported in multiple publications (≧2), consisting of 182 (48.79%) and 191 (51.21%) primary and secondary metabolites, respectively. Among the reported secondary metabolites, 135 were phenolic compounds, accounting for 70.68% of the secondary metabolites (Table S2). In addition, the content of Phe, a precursor of phenolic compounds, varied widely among the various flesh samples, ranging from 6.98 to 405 µg/g (Table S5).

### 3.2 *A. trifoliata* transcriptome sequencing data and gene annotation

A total of nine sets of available raw transcriptome datasets (BioProjects PRJNA685604, PRJNA792843, PRJNA884501, PRJNA671772, PRJCA014987, PRJNA524995, PRJNA1098036, PRJCA045110, and PRJNA857910), comprising 153 cDNA libraries from various tissues, including leaves, peels, flesh, seeds, and whole fruits, were obtained from the corresponding databases or linked publications (Table 1), and the geographical distribution of the transcriptomic samples covered almost all subtropical regions of China where *A. trifoliata* originated and where wild resources were distributed (Figure 1A,B) (Dong et al., 2024). A total of 1149.4 Gb of raw sequencing data from 51 samples with three biological replicates was obtained, generating 1067.46 Gb of clean data by filtering low‐quality and redundant sequences. The size, GC content, and mapping rate of the clean data varied from 5.3 to 20 Gb, from 41% to 48%, and from 68.54% to 94.51%, respectively, among the various samples. Notably, the average mapping rates of three samples (PM, PS, and PL) were clearly lower than those of the remaining 48 samples (Table S3).

**FIGURE 1:**
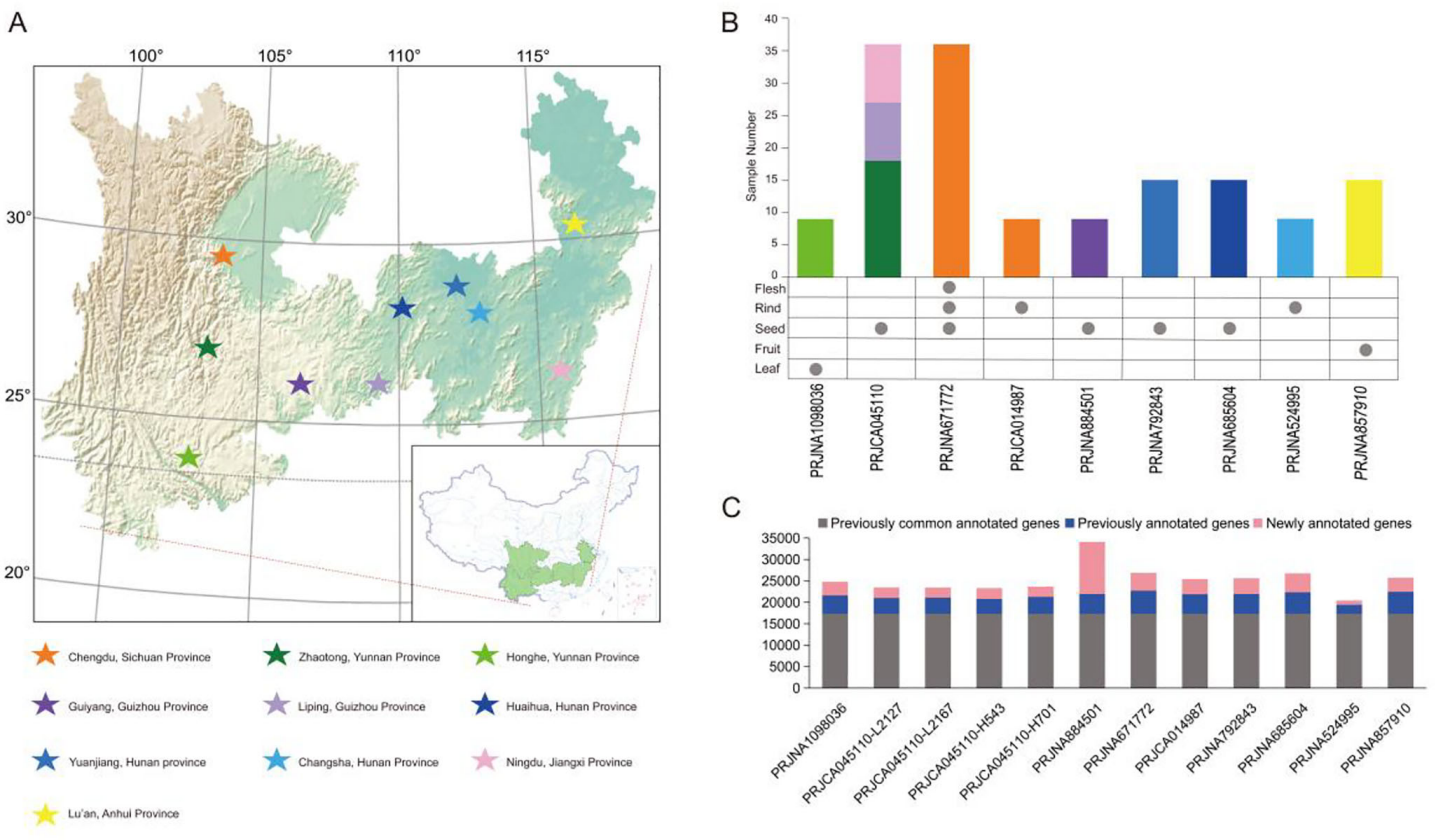
Sources and preliminary analysis of transcriptomic datasets of *Akebia trifoliata*. (A) Geographic distribution of plant samples used for transcriptomic data analysis. (B) Statistics of sample numbers and tissue sources across all transcriptomic datasets. (C) Statistics of previously annotated genes and newly identified genes compared with the reference genome.

The transcriptome dataset from BioProject PRJNA884501 contained the largest number of identified genes (34,102), including 21,959 previously annotated genes and 12,143 newly annotated genes. In contrast, the transcriptome dataset from BioProject PRJNA524995 contained the fewest identified genes (20,423), including 19,425 previously annotated genes and 998 newly annotated genes. Further analysis revealed that 17,340 previously annotated genes were common across all nine transcriptome datasets (Figure 1C; Table S6).

### 3.3 Correlation analysis and detection of sequence variants

Generally, the repeatability of each sample was high, and a strong positive correlation was observed among samples from the same tissues, whereas weak positive correlations were observed among samples from different tissues (Figure 2A; Table S7). For example, correlations among seed samples collected at the same developmental stage were very high, whereas transcript expression patterns in leaf samples usually showed weak correlations with those in other tissues (Figure 2A). Principal component (PC) analysis revealed that PC1 and PC2 separated BioProject PRJNA1098036 (leaf) and BioProject PRJNA884501 (seed) from the other seven BioProjects, respectively, and no overlap was observed between these two BioProjects. Interestingly, BioProject PRJNA671772 (seed) overlapped with the other eight BioProjects except PRJNA1098036, PRJNA884501, and PRJCA045110, whereas only PRJCA014987 (peel) overlapped with BioProject PRJNA671772 (Figure 2B).

**FIGURE 2:**
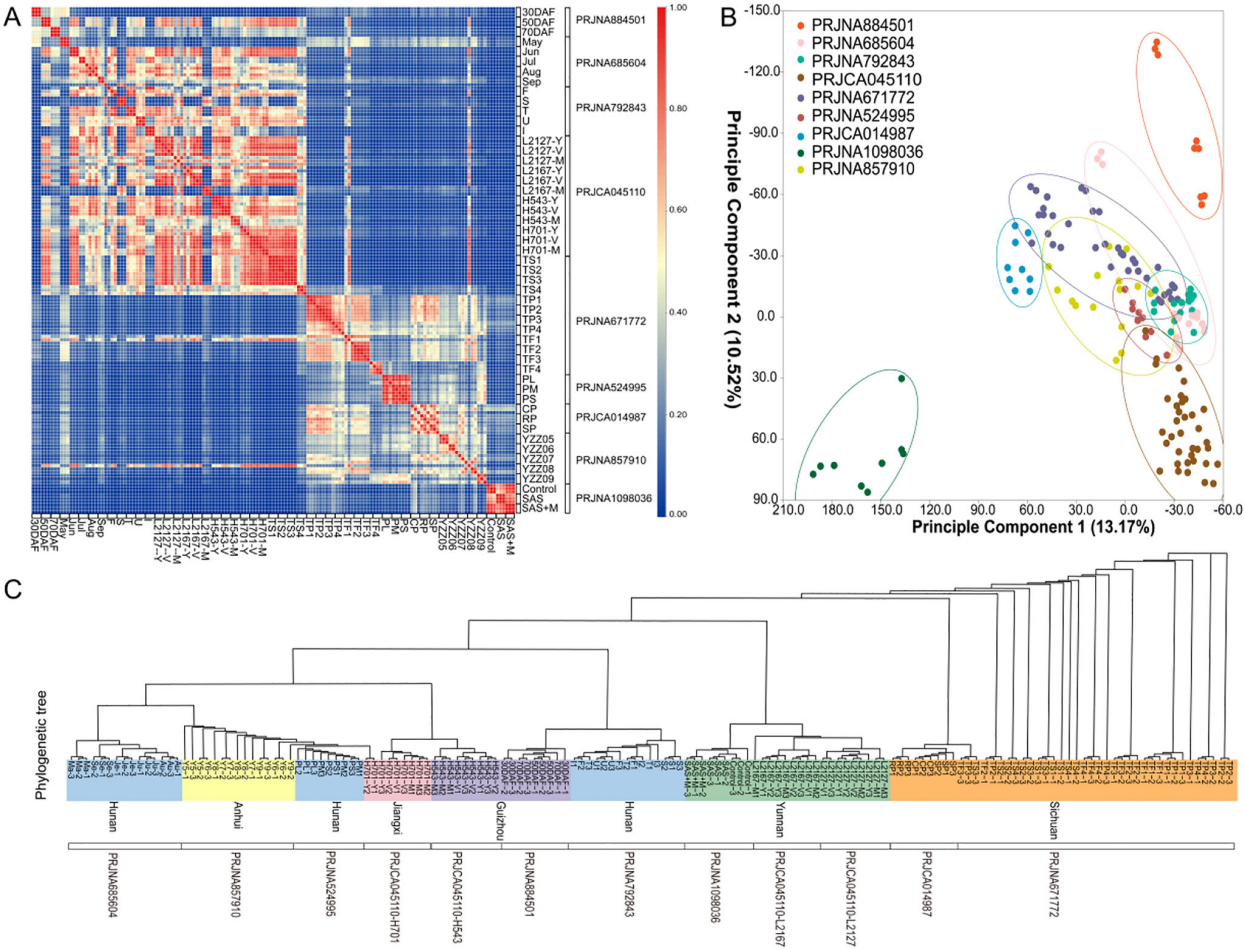
Correlation analysis and principal component analysis of the transcriptome expression of *Akebia trifoliata*. (A) Correlation heatmap of all transcriptome samples. (B) Principal component analysis of all transcriptome samples. (C) Phylogenetic tree of all transcriptomes based on mutation sites.

A total of 12,821,723 variants were identified by sequence comparison, among which 11,908,664 (92.88%) were single‐nucleotide polymorphisms (SNPs), whereas insertion (INS) variants and deletion (DEL) variants accounted for only 526,387 (4.10%) and 386,672 (3.02%), respectively. The average variant density was 20 bp per kb, ranging from 17.5 bp per kb on chromosome 13 to 24.4 bp per kb on chromosome 4. Physically, the variants were unevenly distributed on all 16 chromosomes and unassembled contigs (Table S8). For example, chromosome 3 contained the highest number of variants (1,396,438; 10.89%), whereas chromosome 9 contained the lowest number (504,198; 3.93%).

A phylogenetic tree based on genetic distances was constructed to illustrate the relationships among samples (Figure 2C), and samples from each transcriptomic dataset were clearly clustered together. Among the eight provinces represented by the transcriptomic samples, samples from Sichuan (BioProject PRJNA671772) showed the shortest genetic distance from the reference genome, whereas samples from Hunan (PRJNA685604) showed the greatest genetic distance (Figure 2C).

### 3.4 Expression profile and sequence variants of *AktADTs* in various samples

The expression of *AktADT1*, *AktADT2*, and *AktADT5* was generally low, whereas *AktADT6* showed consistently high expression. The remaining four *AktADT* genes, especially *AktADT3* and *AktADT8*, exhibited obvious stage‐specific expression patterns. In detail, *AktADT3* was highly expressed during the young stages of fruit peel and flesh (TP1 and TF1), while *AktADT8* showed elevated expression at the early stages of seed development (30 DAF, May, June, and HO4‐Y). (Figure 3A). In addition, the expression of *AktADT8* was also related with disease infection (SP) and abiotic stress condition (SAS + M) (Figure 3A; Table S9).

**FIGURE 3:**
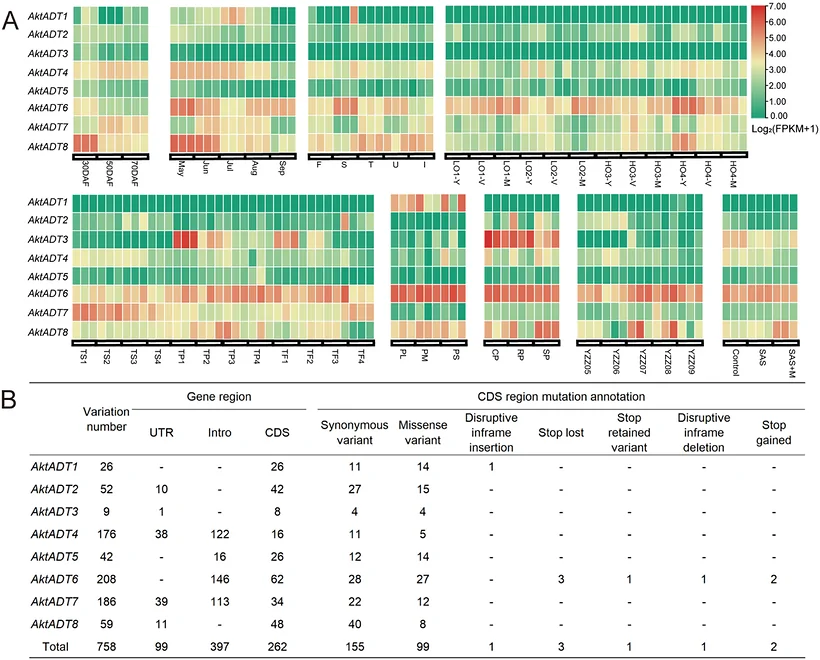
Expression pattern and statistics for variants in *AktADTs* in the *Akebia trifoliata* transcriptome. (A) Expression patterns of the *AktADT* genes in all the transcriptome samples. (B) The distribution and type of all the variant sites in the *AktADT* genes.

In the available transcriptomic data from 51 *A. trifoliata* samples, a total of 758 variant sites consisting of 723 SNPs, 24 INSs, and 12 DELs were detected, in which the variant in the second intron of *AktADT6* on chromosome 12 was a composite type of both a DEL and INS of a thymine “T” (Figure 3B; Table S10). Generally, BioProjects PRJNA884501, PRJNA685604, and PRJNA792843 had noticeably greater numbers of variants than the other datasets, and similarly, the number of variants in various *AktADTs* ranged from 9 in *AktADT3* to 208 in *AktADT6* (Figure 3B).

Physically, 262, 397, and 99 sites were located in the CDSs, introns, and untranslated regions (UTRs), respectively. Further observation revealed that variants occurred in the CDS region of all the *AktADTs* but occurred only in the introns and UTRs of some of the *AktADTs*. Functionally, the variants in the CDS regions of all the *AktADTs* included both synonymous and missense mutations, with the highest numbers observed in *AktADT8* and *AktADT6*, respectively. In contrast, disruptive inframe INSs and both loss and gain of stop codons occurred only in *AktADT1* and *AktADT6*, respectively.

### 3.5 Correlation analysis of *AktADTs* with metabolites and enzyme genes of Phe metabolic pathway

Among the nine transcriptomic datasets, only BioProject PRJNA671772 had available corresponding metabolomic data (S. F. Zhong, Guan, et al., 2022). Therefore, 36 samples from this BioProject were employed to further determine the association network. Correlation analysis identified significant associations between *AktADT* genes and 124 metabolites, including 48 flavones, 28 flavonols, 16 flavanones, 7 flavanols, 7 hydroxycinnamoyl derivatives, 5 anthocyanins, and 13 other metabolites, which were involved in five Phe‐related metabolic pathways, including flavone and flavonol biosynthesis (map00944), phenylpropanoid biosynthesis (map00940), flavonoid biosynthesis (map00941), isoflavonoid biosynthesis (ko00943), and anthocyanin biosynthesis (map00942). Among these metabolites, 15 flavones, 14 flavonols, 5 flavanones, 5 flavanols, 4 hydroxycinnamoyl derivatives, 2 anthocyanins, and 6 other metabolites were reported in multiple publications. Additionally, 150 enzyme‐encoding genes in these pathways were found, and only *AktADT7* and *AktADT8* were significantly correlated (*p *< 0.01) with both the metabolites and genes in each pathway. Interestingly, only *AktADT8* was significantly positively correlated with 49 genes and 34 metabolites across these pathways, with 13 of these metabolites being reported in multiple publications (Figure 4; Tables S11 and S12).

**FIGURE 4:**
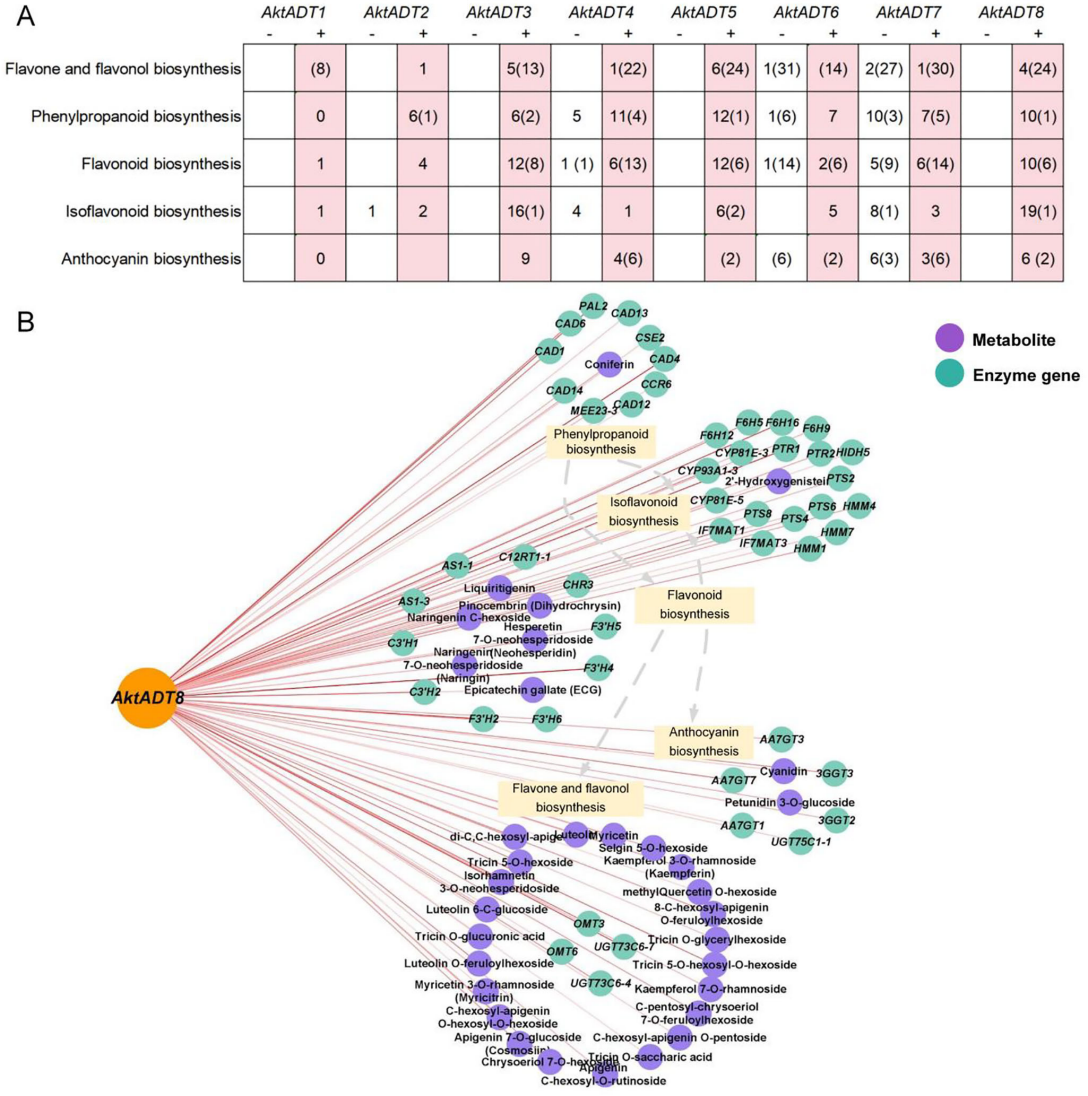
Correlation between *AktADT* expression and enzymes‐encoding genes and metabolites involved in the Phe metabolic pathway. (A) Statistics of the correlations between *AktADT* expression and metabolite levels and enzyme‐encoding gene expression in the Phe metabolic pathway; “−” indicates the number of negative correlations, “+” indicates the number of positive correlations, and brackets indicate the number of metabolites. (B) Correlation network between the expression of *AktADT8* and the expression of genes encoding enzymes and metabolites of the Phe metabolic pathway.

### 3.6 Phenotyping of *A. thaliana* overexpressing *AktADTs*

To understand the functions of the *AktADT* genes, the coding regions of these genes were inserted into the pCAMBIA1305‐Gus vector by homologous recombination, and the recombinant pCAMBIA1305‐AktADTs‐GUS vectors were transfected into *A. tumefaciens* GV3101 and used to infect *A. thaliana* (Figure 5A–C). *AktADT5* overexpressing plants showed significant abnormalities in both leaf and whole‐plant development, exhibiting curled rosette leaves with serrated edges and delayed development (Figure 5C), whereas in *AktADT8* overexpressing plants, shoot development and flowering occurred significantly earlier than in the other lines (Figure 5D). In addition, *AktADT6* and *AktADT8* overexpressing plants exhibited significantly higher Phe and flavonoid contents compared with wild‐type plants (*p* < 0.05); notably, the Phe content was almost doubled in the *AktADT8*‐overexpressing plants (Figure 5E,F).

**FIGURE 5:**
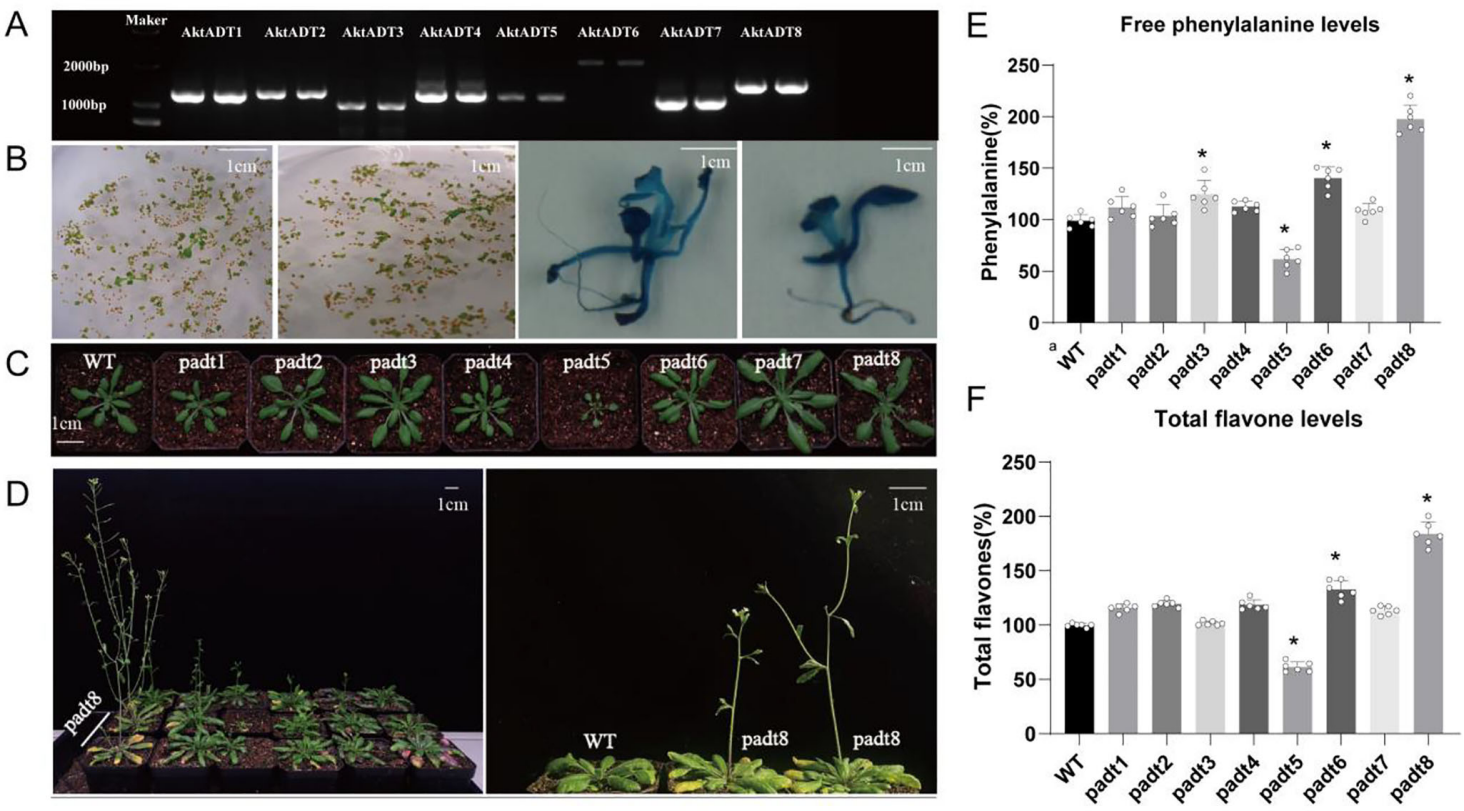
Identification and phenotypic observation of the AktADT‐overexpressing lines. (A) Polymerase chain reaction (PCR) amplification of the *AktADTs* of *Akebia trifoliata*. (B) Identification of positive seedlings from the overexpression lines. (C) Overall growth phenotypes of the overexpression lines. (D) Early flowering and bolting of the *AktADT8* overexpressing lines. (E) Phe content in the leaves of the overexpression and wild type (WT) lines; the content in the overexpression lines is reported relative to WT lines, in which the value was set to 100%, and asterisk (*) indicates a significant difference at *p* < 0.05. (F) Total flavonoid content in the leaves of the overexpression lines and WT lines; the content in the overexpression lines is reported relative to that in the WT lines, which was set to 100%, and * indicates a significant difference at *p* < 0.05.

### 3.7 Hypotheses on the regulatory network of *AktADT8* and *AktADTs8*

*AktADT8* had two TSS1 and TSS2, and an analysis of the reads of the two corresponding transcripts revealed that the read number of *AktADT8* was greater than that of *AktADTs8* in all 51 samples, especially the TS4, TR4, TF4, YZZ08, and SAS + M samples when compared in the same project (Figure 6A; Table S13).

**FIGURE 6:**
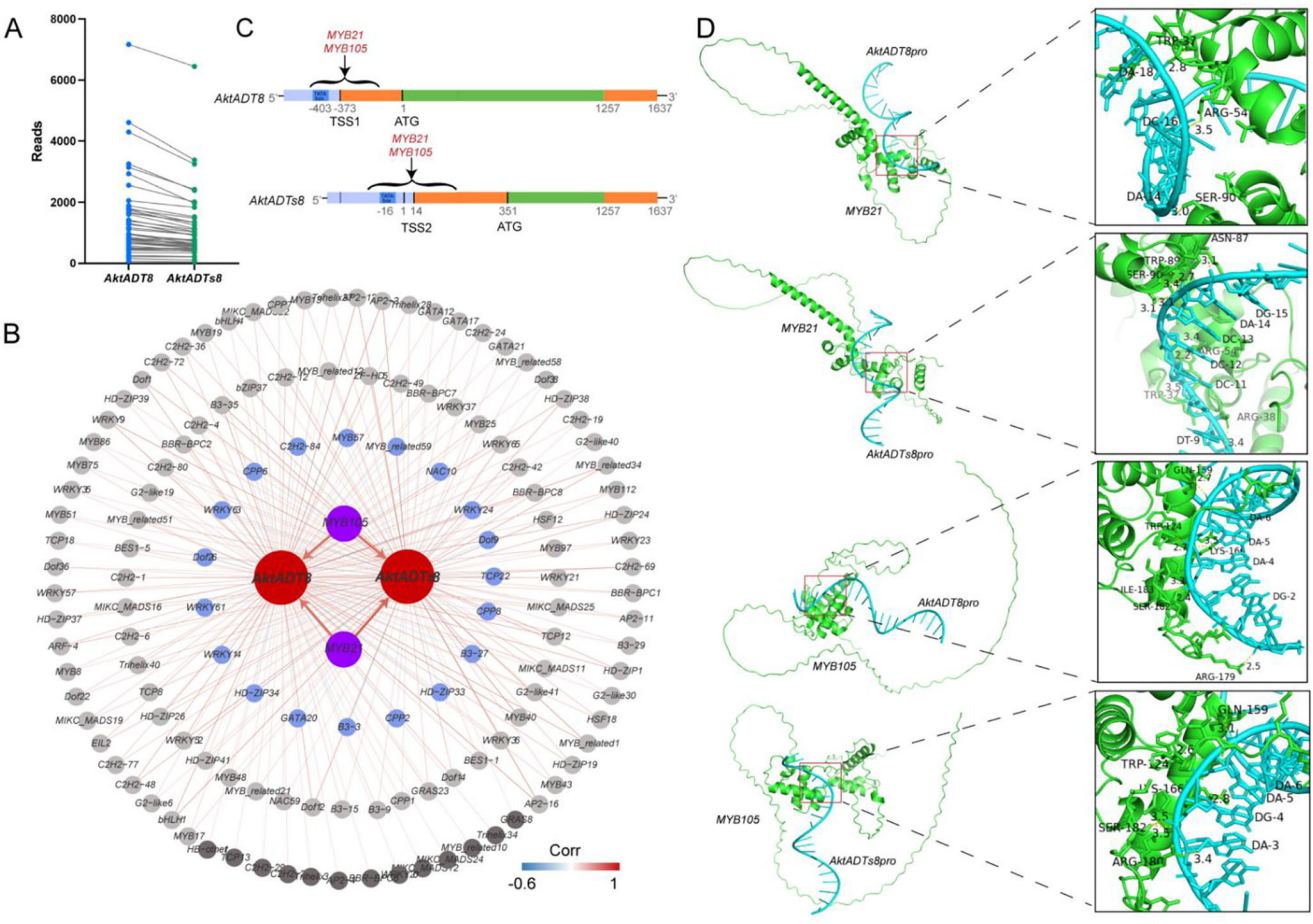
Expression and hypotheses of regulatory networks of the *AktADT8* and *AktADTs8* transcripts in *Akebia trifoliata*. (A) Read count statistics for *AktADT8* and *AktADTs8*. (B) Co‐expression network of *AktADT8* and transcription factors; arrows indicate TFs predicted to bind to gene promoters; red arrows indicate significant positive correlations (*p* < 0.05); red circles indicate the two target transcripts; purple circles indicate selected TFs significantly positively correlated with both transcripts; gray circles indicate TFs that were significantly positively correlated with *AktADT8* and *AktADTs8* expression; blue circles indicate TFs that were significantly negatively correlated with *AktADT8* and *AktADTs8* expression; and dark gray circles indicate TFs that were significantly positively correlated with only *AktADT8* (*p *< 0.05). (C) Binding sites for transcription factors in the *AktADT8* and *AktADTs8* promoter regions. (D) Molecular docking simulations of MYB21 and MYB105 (where MYB is v‐myb avian myeloblastosis viral oncogene homolog) proteins with the predicted sequences in the core promoter region.

The results for the predicted TFBSs showed that the promoter regions of *AktADT8* and *AktADTs8* contained putative binding sites for 286 and 243 TFs, respectively, and 231 TFs were common between the two promoters (Table S14). The sequence analysis revealed that the TATA box cis‐acting element was present 30 bp upstream of TSS1 and TSS2 (Table S15). Among the TFs, *MYB105* and *MYB21* were predicted to bind to the core promoter regions of *AktADT8* and *AktADTs8*, which were located near TSS1 and TSS2, respectively. Co‐expression analysis showed that both *MYB21* and *MYB105* were significantly positively correlated with the expression of *AktADT8* and *AktADTs8* (Tables S14 and S15; Figure 6B,C). In addition, a total of 134 and 120 TFs exhibited significant (*p *< 0.05) correlations with *AktADT8* and *AktADTs8* expression, respectively (Figure 6B). Among these, 119 TFs simultaneously had positive relationships with the expression of both *AktADT8* and *AktADTs8*, whereas 18 TFs had negative relationships with both genes. Notably, 15 TFs were significantly positively correlated only with *AktADT8* expression, whereas 1 TF (*GRAS8*) was significantly positively correlated only with *AktADTs8* expression (Figure 6B; Table S16).

Finally, the predicted results obtained through molecular docking of two TFs and the predicted sequences in the core promoter region (Table S15) are shown in Figure 6D. The TRP at position 37, ARG at position 54, and SER at position 90 of the MYB21 protein formed hydrogen bonds with the A base at position +114 bp of TSS1, the C base at position +112 bp of TSS1, and the A base at position +110 bp of TSS1 in *AktADT8*, respectively, with hydrogen bond lengths ranging from 2.8 to 3.5 angstroms (Å). The MYB21 protein showed potential binding to the predicted base sequence of *AktADTs8* (−24 to −18 bp of TSS2), with nine hydrogen bond sites showing distances ranging from 2.2 to 3.5 Å. Similarly, the MYB105 protein showed six interaction sites with the predicted base sequence of *AktADT8* (+68 ∼ +72 bp of TSS1), with hydrogen bond distances ranging from 2.4 to 3.5 Å. The MYB105 protein also showed six interaction sites with the predicted base sequence of *AktADTs8* (+215 ∼ +218 bp at TSS2), with hydrogen bond distances ranging from 2.6 to 3.5 Å.

### 3.8 *MYB21* and *MYB105* differentially regulate *AktADT8* and *AktADTs8* through direct promoter binding

To investigate the regulatory effects of *MYB21* and *MYB105* on *AktADT8pro* and *AktADTs8pro*, a dual‐luciferase reporter assay system was first performed. Compared with the corresponding negative controls, co‐expression of *MYB21* with *AktADT8pro*, *MYB105* with *AktADT8pro*, and *MYB21* with *AktADTs8pro* produced intense luminescence signals, whereas co‐expression of *MYB105* with *AktADTs8pro* resulted in no appreciable change in luminescence (Figure 7A). Dual‐luciferase reporter assays further showed that both *MYB21* and *MYB105* significantly enhanced *AktADT8* promoter activity to approximately 2.0‐fold of the control level. *MYB21* significantly increased *AktADTs8* promoter activity to approximately 1.5‐fold the control level, whereas *MYB105* had no significant effect on the activity of this promoter (Figure 7B). Yeast one‐hybrid (Y1H) assays further demonstrated that both *MYB21* and *MYB105* bound to *AktADT8pro* under selection conditions containing 50 ng/mL AbA. In addition, at an AbA concentration of 900 ng/mL, *MYB21* bound to *AktADTs8pro*, whereas no detectable binding of *MYB105* to this promoter was observed (Figure 7C). Collectively, these results demonstrate that *MYB21* directly binds to and activates the promoters of both *AktADT8* and *AktADTs8*, whereas *MYB105* specifically binds to and activates the *AktADT8* promoter.

**FIGURE 7:**
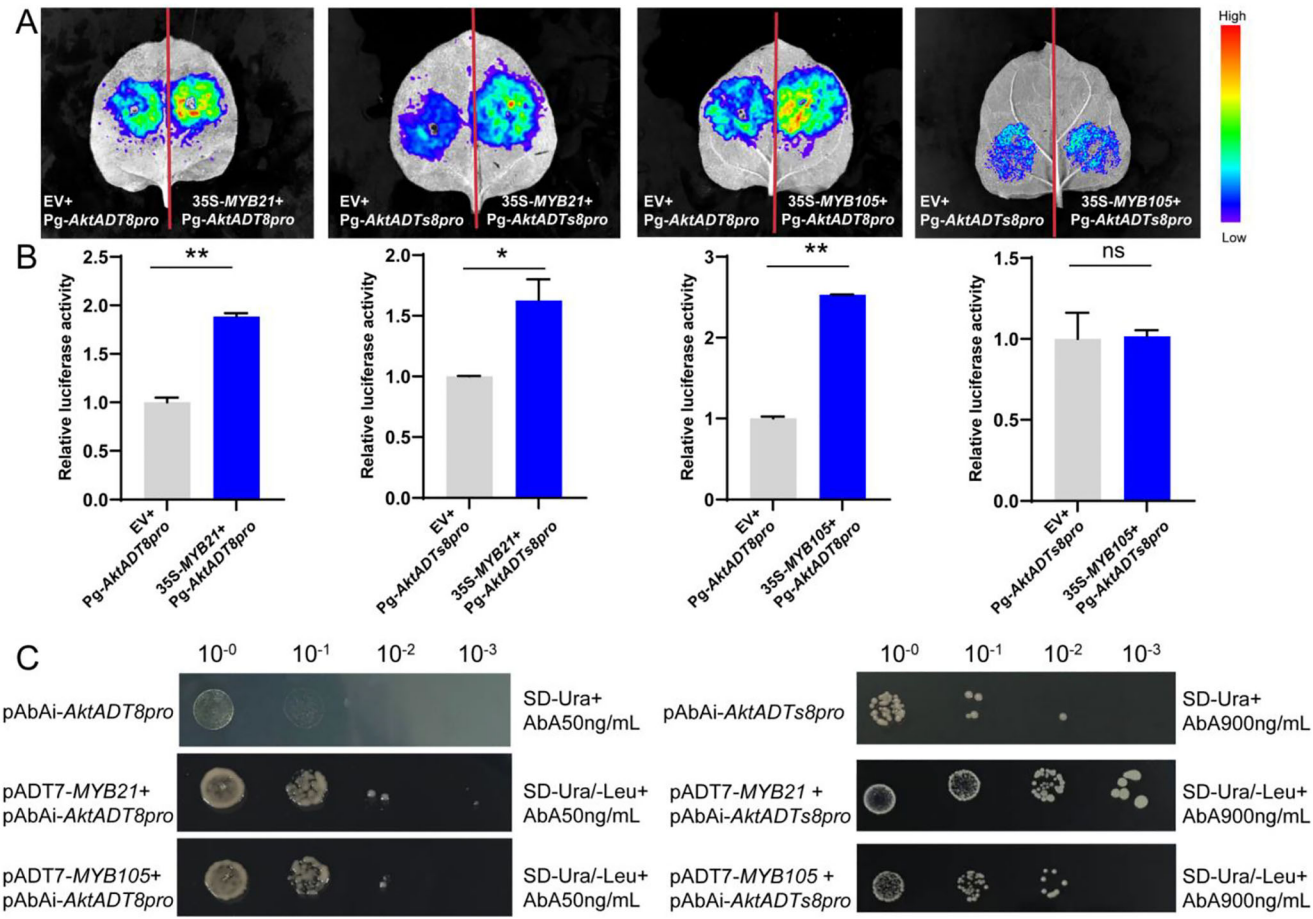
Differential regulation of *AktADT8pro* and *AktADTs8pro* by *MYB21* and *MYB10*5 (where MYB is v‐myb avian myeloblastosis viral oncogene homolog). (A) Luciferase complementation imaging assays showing the interactions of *MYB21* and *MYB105* with *AktADT8pro* and *AktADTs8pro*. EV indicates the empty pCAMBIA1301‐35S vector. The pseudocolor scale indicates luminescence intensity, which increases from purple to red. (B) Dual‐luciferase reporter assays showing the effects of *MYB21* and *MYB105* on the promoter activities of *AktADT8* and *AktADTs*8. Data in bar graphs are presented as mean ± SD (*n* = 3 biological replicates). Asterisks indicate significant differences by two‐tailed Student's *t*‐test (*p < 0.05, **p < 0.01, and ns indicates no significant difference). (C) Yeast one‐hybrid (Y1H) assays showing the binding of *MYB21* and *MYB105* to the *AktADT8pro* and *AktADTs8pro*.

## 4 DISCUSSION

### 4.1 Phenolic compounds could contribute to the health value of *A. trifoliata*

Plants possess endogenous flavonoid biosynthetic pathways, specialized subcellular compartments, and well‐established genetic engineering systems, making them ideal hosts for the efficient biosynthesis of flavonoids (Y. Xu, Ge, et al., 2025). Therefore, systematically elucidating the biosynthesis and regulatory mechanisms of plant bioactive compounds is important for improving their natural accumulation and facilitating their utilization. In this study, 191 secondary metabolites were identified in *A. trifoliata*, accounting for 51.21% of the metabolites reported across multiple publications (Table S2). Among these secondary metabolites, the number of phenolics was 135 (70.68%), which was far greater than the numbers of terpenoids and alkaloids (Table S2). These findings suggested that the medicinal and nutritional value of *A. trifoliata* may be largely associated with the abundant phenolic compounds, especially flavonoids, rather than terpenoids and alkaloids. This profile differs from those of other medicinal plants, such as *Panax ginseng*, whose bioactive properties are largely associated with terpenoids (R. Wang et al., 2025), and *Aconitum carmichaelii*, whose medicinal properties are mainly attributed to alkaloids (Zhao et al., 2023).

Previous studies have shown that the content of Phe, an important precursor, is far higher in *A. trifoliata* than in common fruits such as apple, banana, grape, and pear (S. F. Zhong, Guan, et al., 2022). Here, the reported Phe contents in *A. trifoliata* fruits were generally high, although substantial variation was observed among different samples (Table S5). This variation may reflect differences in genetic background, developmental stage, or environmental conditions and may be associated with the potentially active Phe biosynthetic capacity reported for *A. trifoliata*.

### 4.2 *AktADT8* could play a pivotal role in regulating the biosynthesis of Phe and its derivatives

Previous studies have demonstrated that ADTs strongly influence the biosynthetic process of Phe and its downstream metabolism by regulating both the rate of Phe production and its subsequent metabolic allocation (Maeda et al., 2010; Qian et al., 2019). A recent study found eight *AktADT* genes in the *A. trifoliata* reference genome, among which *AktADT1*, *AktADT4*, *AktADT7*, and *AktADT8* encode proteins exhibiting PDT activity in addition to ADT activity (Guan et al., 2024). In this study, the distribution of 51 samples covered most of the wild distribution areas of *A. trifoliata* (Table 1; Figure 1A) (Dong et al., 2024). The distinct expression patterns of *AktADT4*, *AktADT7*, and *AktADT8* suggest that these genes may perform specialized functions during development and stress responses (Figure 3A). Similar developmental and stress‐responsive expression patterns have also been reported for *ADT* genes in *Zea mays* (Ren et al., 2024). Interestingly, the accumulation of Phe and several of its derivatives, especially flavonoids, also exhibited obvious stage‐specific or stress‐specific patterns (C. Chen, Zhou, et al., 2025; H. Yang, Zhong, et al., 2024). These findings suggest a potential association that *AktADT4*, *AktADT7*, and *AktADT8* could be involved in the biosynthesis of Phe and its derivatives.

Synonymous variations can influence gene expression and ultimately affect plant functions by altering RNA splicing, RNA modification, RNA secondary structure, translation efficiency, and cis‐regulatory elements within coding regions (M. Liu et al., 2026). In addition, genetic variation represents an important basis for plant adaptation to diverse environments by shaping key metabolic pathways and adaptive traits (B.‐J. Chen, Wang, et al., 2025). The relatively high level of synonymous variation observed in the CDS region of *AktADT8* may reflect the evolutionary diversity associated with Phe synthesis and environmental adaptation (Figure 3B). Transcriptomic analysis further indicated that the expression of *AktADT8* was also associated with infection status and abiotic stress exposure (Figure 3A). These results provide a reasonable explanation for the environmental adaptation of *A. trifoliata*, because Phe derivatives such as flavonoids usually increase the defense responses to biotic and abiotic stresses (Y. Xu, Ge, et al., 2025; H. Yang, Wang, et al., 2024). Previous studies of subcellular localization suggested that the three *AktADTs* were localized in chloroplasts, where the arogenate pathway of Phe synthesis occurs, but the alternative (*AktADTs8*) produced by expression through the use of one of two alternative TSSs was located in the cytosol, where the phenylpyruvate pathway of Phe synthesis occurs (Figure 6B) (Guan et al., 2024; Qian et al., 2019). These findings suggest that *AktADT8* may contribute to Phe biosynthesis through both the arogenate and phenylpyruvate pathways, thereby providing potential metabolic flexibility under different developmental and environmental conditions.

Functionally, the enhanced accumulation of Phe and flavonoids in AktADT8‐overexpressing plants supports a positive role for *AktADT8* in promoting metabolic flux toward phenylpropanoid biosynthesis (Figure 5C–F). This observation is consistent with previous studies showing that overexpression of *AtADT4* and *AtADT5* similarly promotes Phe accumulation and downstream metabolism in *A. thaliana* (Q. Chen et al., 2016), suggesting that the regulatory functions of *ADTs* may be evolutionarily conserved. Notably, *AktADT8* belongs to the γ‐type *ADT* subgroup, whose members generally maintain higher catalytic activity and are less sensitive to feedback inhibition of Phe than α‐ or β‐type *ADTs* (Guan et al., 2024; Li et al., 2024). These characteristics may explain the pronounced effects of *AktADT8* on Phe accumulation and flavonoid biosynthesis observed in this study. In addition, the co‐expression analysis revealed that the expression of *AktADT8* was significantly positively correlated with 49 enzyme‐coding genes and 34 metabolites involved in five flavonoid biosynthesis pathways, with 13 of these metabolites reported in multiple publications, highlighting their potential importance for future pathway analysis (Figure 4). This regulation of biosynthetic pathways may contribute to the early bolting and flowering in the *AktADT8* overexpression line (Figure 5D). Taken together, these findings suggest that *AktADT8* may represent an important candidate gene in the phenylpropanoid pathway of *A. trifoliata*, not only regulating Phe biosynthesis by influencing the components and content of its derivatives, especially flavonoids. However, further functional and mechanistic studies are required to confirm its precise regulatory role within this pathway.

### 4.3 Putative regulatory network of *AktADT8* in *A. trifoliata*

Alternative transcription initiation contributes to remarkable diversity and flexibility in gene expression (X. Wang, Duan, et al., 2024). Previous studies have shown that, among the *ADT* family members expressed in *A. trifoliata*, only the *AktADT8* gene contains two TSS1 and TSS2 that exhibited alternative transcription patterns (Guan et al., 2024). In this study, the transcriptomic data generated further confirmed the expression of both transcripts, with *AktADT8* consistently exhibiting higher transcript abundance than *AktADTs8* (Figure 6A), supporting the occurrence of alternative transcription initiation in this gene.

Alternative TSS can produce transcripts with different N‐terminal sequences, potentially affecting protein structure, subcellular localization, and functional regulation (Ly et al., 2025). Previous studies have shown that *AktADT8* localizes to the chloroplast, whereas *AktADTs8* localizes to the cytoplasm and may play different metabolic roles in the Phe biosynthetic pathway (Guan et al., 2024). Although the core catalytic regions (such as the PAC domain) are conserved between the two transcripts, N‐terminal variations may affect enzymatic regulation, protein‐protein interactions, and the control of metabolic flux (Tong et al., 2025). Transcriptomic analysis showed that *AktADT8* expression was consistently higher than *AktADTs8* across all samples, but in several specific stages or conditions (e.g., SAS + M, TF4, TR4, and TS4), the expression of *AktADTs8* was further reduced (Figure 6A). This expression pattern suggests that the auxiliary role of *AktADTs8* may be attenuated, or the contribution of *AktADT8* is enhanced under certain specific conditions. Research has shown that chloroplasts are the key regulatory centers of plant immunity and play central role in activating immune responses and balancing plant growth and defense (Rui et al., 2025). When considered together with the distinct subcellular localizations of the two transcripts, it is plausible that chloroplast‐localized *AktADT8* may contribute to Phe metabolism under specific environmental conditions. However, this hypothesis remains speculative and requires further experimental validation.

TFs are the key trans‐acting proteins that regulate gene expression (Duttke et al., 2024; Kidokoro et al., 2023), whereas TATA‐boxes in the promoter region are key cis‐acting elements for determining the initiation and rate of gene expression (Butler & Kadonaga, 2001; Cui et al., 2023). Promoter structure analysis revealed the presence of TATA boxes at approximately −30 bp relative to both TSS1 and TSS2. TSSs are essential components of promoters and are usually clustered into transcription start regions (TSRs) (Policastro et al., 2020). Canonical TSRs are generally located within the core promoter regions (X. Wang, Duan, et al., 2024), which typically encompass approximately 40 bp upstream and downstream of the TSS, although their precise boundaries vary among species and genes (Vo Ngoc et al., 2020). Binding prediction analysis indicated that *MYB21* and *MYB105* could potentially bind to the core promoter regions of *AktADT8* and *AktADTs8* (Table S15). Furthermore, correlation analysis showed that the expression levels of both TFs were significantly positively correlated with those of *AktADT8* and *AktADTs8* (Table S16; Figure 6B). Dual‐luciferase reporter assays and Y1H assays further demonstrated that *MYB21* binds to the promoter regions of both transcripts and activates their promoter activities, whereas *MYB105* specifically binds to and activates the core promoter region of *AktADT8* (Figure 7). Binding site prediction suggested that the MYB105 binding sites associated with *AktADTs8* were located relatively far from TSS2 and may not be within the core promoter region, which could explain the lack of regulatory activity toward the *AktADTs8* promoter. Interestingly, previous studies have shown that *MYB4* represses *ADT6* expression in *Arabidopsis*, thereby reducing flavonoid accumulation, and that *MYB* TFs can coordinately regulate alternative ADT isoforms with distinct subcellular localizations (X. C. Wang et al., 2020). Compared with many common fruit crops, *A. trifoliata* contains significantly higher levels of Phe (S. F. Zhong, Guan, et al., 2022) and abundant flavonoids (H. Yang, Zhong, et al., 2024), suggesting that ADT‐mediated Phe biosynthesis may exhibit high activity in *A. trifoliata*. Therefore, *MYB21* TFs may function as positive regulators of *AktADT8* and *AktADTs8* in *A. trifoliata*. Collectively, the prediction and experimental evidence presented here suggest that *MYB21* was involved in the complex regulation of *AktADT8* and *AktADTs8* expression. However, due to the current limitations of the genetic transformation system in *A. trifoliata*, further studies are required to obtain additional evidence and fully elucidate the regulatory mechanisms underlying ADT‐mediated Phe and flavonoid biosynthesis.

## 5 CONCLUSIONS

Figure 8 summarizes the main findings of this study. Here, 135 Phe‐derived polyphenol compounds were identified, accounting for 70.68% of the secondary metabolites reported in multiple publications, which may benefit the medicinal value and environmental adaptability of *A. trifoliata*. Genetically, the rich polyphenols may be associated with *AktADT8* function, which is involved in the biosynthetic pathway of Phe, as supported by the phenotypic changes observed in the overexpression lines of *Arabidopsis*, expression profiles, sequence variants, and co‐expression network analysis. Functionally, 135 TFs were predicted to bind to the promoter regions of *AktADT8* and *AktADTs8* and showed significant associations with their expression patterns. Among them, *MYB21* was experimentally demonstrated to activate the transcriptional activity of both *AktADT8* and *AktADTs8*, whereas *MYB105* specifically activated *AktADT8*. These findings provide a versatile strategy for enhancing the biosynthetic pathway of Phe and its derivatives.

**FIGURE 8:**
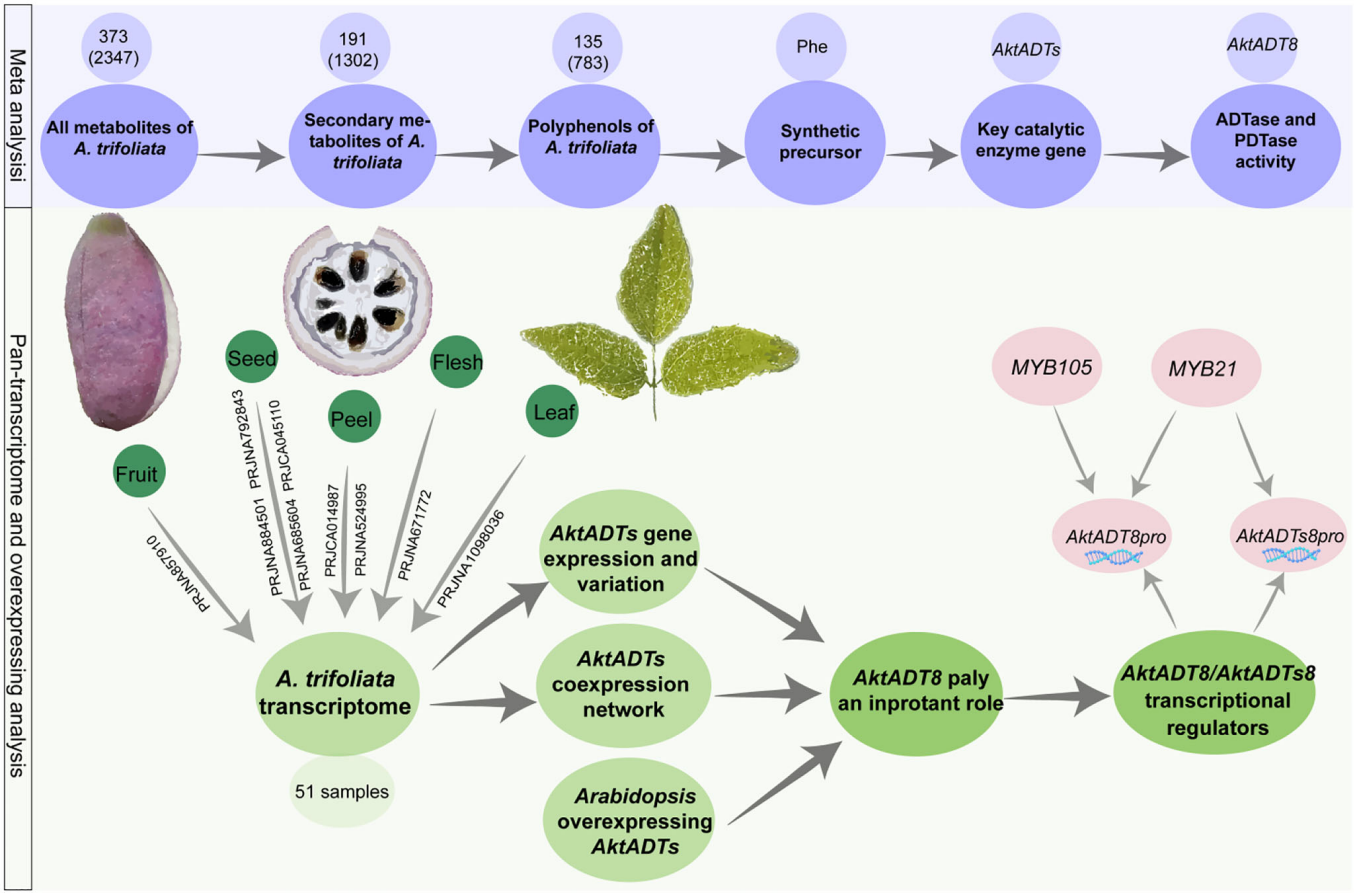
Schematic diagram of the research process and results.

## AUTHOR CONTRIBUTIONS

**Jie Li**: Conceptualization; data curation; formal analysis; investigation; methodology; writing—original draft. **Ju Guan**: Data curation; investigation; methodology; validation. **Hao Liu**: Formal analysis; software. **Rui Han**: Data curation; methodology. **Huai Yang**: Formal analysis; resources. **Xiaoxiao Yi**: Data curation; visualization. **Chen Chen**: Methodology; resources. **Feiquan Tan**: Investigation. **Wanyun Peng**: Supervision. **Zhengwei Xie**: Funding acquisition; project administration. **Peigao Luo**: Conceptualization; funding acquisition; supervision; writing—review and editing.

## CONFLICT OF INTEREST STATEMENT

The authors declare no conflicts of interest.

## Supporting information



Supplementary Material

## Data Availability

The datasets supporting the conclusions of this article are available in the CNCB (https://www.cncb.ac.cn/) and NCBI (https://www.ncbi.nlm.nih.gov/) repositories as BioProjects PRJNA671772 (S. F. Zhong, Guan, et al., 2022), PRJNA1098036 (K. Wang, Tao, et al., 2024), PRJNA524995 (Niu et al., 2020), PRJNA884501 (H. J. Liu et al., 2023), PRJNA685604 (H. Huang et al., 2021), PRJCA014987 (H. Yang, Wang, et al., 2024), PRJNA857910 (Fang et al., 2024), PRJCA045110 (Han et al., 2025), and PRJNA792843 (Y. C. Zhong, Zhao, et al., 2022). The data supporting the findings of this study are provided in the Supporting Information.
